# Decreased Circulating Visfatin Is Associated with Improved Disease Activity in Early Rheumatoid Arthritis: Data from the PERAC Cohort

**DOI:** 10.1371/journal.pone.0103495

**Published:** 2014-07-28

**Authors:** Ondřej Sglunda, Heřman Mann, Hana Hulejová, Markéta Kuklová, Ondřej Pecha, Lenka Pleštilová, Mária Filková, Karel Pavelka, Jiří Vencovský, Ladislav Šenolt

**Affiliations:** 1 Institute of Rheumatology, Prague, Czech Republic; 2 Department of Rheumatology, First Faculty of Medicine, Charles University in Prague, Prague, Czech Republic; 3 Technology Centre ASCR, Prague, Czech Republic; SERGAS, Santiago University Clinical Hospital, IDIS Research Laboratory 9, NEIRID Lab, Spain

## Abstract

**Objective:**

To evaluate circulating visfatin and its relationship with disease activity and serum lipids in patients with early, treatment-naïve rheumatoid arthritis (RA).

**Methods:**

Serum visfatin was measured in 40 patients with early RA before and after three months of treatment and in 30 age- and sex-matched healthy individuals. Disease activity was assessed using the Disease Activity Score for 28 joints (DAS28) at baseline and at three and 12 months. Multivariate linear regression analysis was performed to evaluate whether improved disease activity is related to serum visfatin or a change in visfatin level.

**Results:**

Serum visfatin was significantly elevated in early RA patients compared to healthy controls (1.92±1.17 vs. 1.36±0.93 ng/ml; p = 0.034) and significantly decreased after three months of treatment (to 0.99±0.67 ng/ml; p<0.001). Circulating visfatin and a change in visfatin level correlated with disease activity and improved disease activity over time, respectively. A decrease in visfatin after three months predicted a DAS28 improvement after 12 months. In addition, decreased serum visfatin was not associated with an improved atherogenic index but was associated with an increase in total cholesterol level.

**Conclusion:**

A short-term decrease in circulating visfatin may represent an independent predictor of long-term disease activity improvement in patients with early RA.

## Introduction

Visfatin was originally discovered and named pre-B-colony enhancing factor [1] but was later renamed visfatin, reflecting its predominant secretion by visceral adipose tissue [2]. However, visfatin is also produced by several types of immune cells and synovial fibroblasts [3], [4]. Recent studies have demonstrated the involvement of visfatin in innate immunity and inflammation [5], particularly in rheumatoid arthritis (RA). Visfatin is strongly up-regulated in the RA synovial lining layer and at sites of joint invasion, promoting synovial fibroblast motility and increasing the production of pro-inflammatory cytokines and matrix degrading enzymes by synovial fibroblasts and monocytes [3], [6], [7].

In RA, persistent synovial inflammation and invasive behaviour by activated synovial fibroblasts contribute to joint damage, leading to disability [8]. The discovery of novel molecules has contributed to a better understanding of RA pathogenesis and may lead to the identification of biomarkers that would allow for the monitoring of disease activity and individualising prognosis in RA patients [9]. Visfatin may represent a novel biomarker for disease severity. Some data indicate that visfatin is elevated in RA and may be associated with the degree of inflammation, clinical disease activity and radiographic joint damage [3], [10], [11]. However, these findings are not consistent throughout all studies [12]–[14].

The objective of the present study was to characterise 1) the association between serum visfatin level and disease activity in early RA, 2) the effect of treatment with conventional synthetic disease modifying drugs (csDMARDs) on the visfatin level and 3) the relationship between visfatin level and serum lipids.

## Methods

### Patients

A total of 40 patients (28 women) with early RA were included in the study. The inclusion criteria were as follows: 1) age >18 years, 2) fulfilment of the ACR/EULAR 2010 classification criteria for RA at baseline [15], 3) symptom duration of ≤6 months and 4) no or only symptomatic therapy with nonsteroidal antirheumatic drugs at baseline. Patients were prospectively followed in the Prague Early RA Clinic (PERAC) at the Institute of Rheumatology in the Czech Republic. The disease activity was assessed using the 28-joint count Disease Activity Score (DAS28-ESR). The control group consisted of 30 age- and sex-matched healthy individuals. Consent procedure was approved by the Ethics Committee of the Institute of Rheumatology. Each participant provided written informed consents prior to entering the study. Both original consents were given to the patients and documented in the patient's files.

### Laboratory analysis

Fasting blood samples were collected from all patients at baseline and after three months. The samples were immediately centrifuged and stored at −20°C. The serum concentration of visfatin was measured using a commercially available enzyme-linked immunosorbent assay (ELISA) (Biovision, Milpitas, California, USA) as described previously [14]. The levels of serum anti-cyclic citrullinated peptide antibodies (anti-CCP) and IgM rheumatoid factor (IgM-RF) were measured using a standard ELISA assay (Test Line S.R.O., Czech Republic). CRP and total and HDL-cholesterol were determined using routine laboratory techniques.

### Statistical analysis

Pearson's and Spearman's rank correlations were used in cases of normal and non-normal distributions. The T-test was used for normal variables, and the Mann-Whitney test was used for non-parametric variables. Multivariate linear regression analysis was performed to assess the influence of visfatin (its change) on disease activity and blood lipids. P values of less than 0.05 were considered to be statistically significant. Statistical analyses were performed using SPSS 17 (SPSS Inc., Chicago, IL, USA) and GraphPad Prism 5.0 (GraphPad Software, Inc., San Diego, CA, USA). The data are presented as the median [range] or mean and standard deviation (SD) in the case of abnormal or normal distribution.

## Results

### Patients and demographic data

Table 1 shows the baseline characteristics of patients and healthy controls included in the study. Twenty patients met the criteria for highly active disease (DAS28>5.1), 18 patients had moderate disease activity (3.2<DAS28≤5.1), and two patients had low disease activity (2.6<DAS28≤3.2) at baseline. Treatment with csDMARDS was initiated in 38 patients at baseline: 31 patients on methotrexate (mean weekly dose at month three was 15 mg; range, 10–20 mg), six on sulphasalazine and one on leflunomide. Additionally, 34 patients were receiving glucocorticoids (mean daily dose at month three was 5 mg; range, 1.25–15.0 mg of prednisone or equivalent per day). Two patients were initially only on glucocorticoids either due to planned pregnancy or elevated aminotransferases. After three months of treatment, a significant reduction in disease activity was observed (DAS28: from 5.3±1.5 to 2.8±1.3; CRP: from 7.7 [0.6–77.8] to 2.3 [0.2–14.3]; p<0.001 for all comparisons).

**Table 1:** Baseline characteristics of patients with early rheumatoid arthritis (RA) and healthy controls.

Characteristics	Early RA	Healthy controls
	(n = 40)	(n = 30)
Gender (F/M)	22/18	19/11
Age (years)	52±17	48±16
BMI	24±4.0	25±3.1
CRP (mg/l)	7.7 [0.6–77.8]	-
DAS28 score	5.25±1.50	-
RF positivity, n (%)	24 (60)	-
Anti-CCP positivity, n (%)	21 (53)	-
Drugs (csDMARDs/GC)	38/31	-

Anti-CCP, anti-cyclic citrullinated peptide antibody; RF, rheumatoid factor; CRP, C-reactive protein; DAS, disease activity score; csDMARDs, conventional synthetic disease modifying antirheumatic drugs; GC, glucocorticoids; RA, rheumatoid arthritis; F, female; M, male; MTX, methotrexate. The data are expressed as a mean (SD) or median [range].

### Visfatin is elevated in early RA and correlates with disease activity

**Figure 1:**
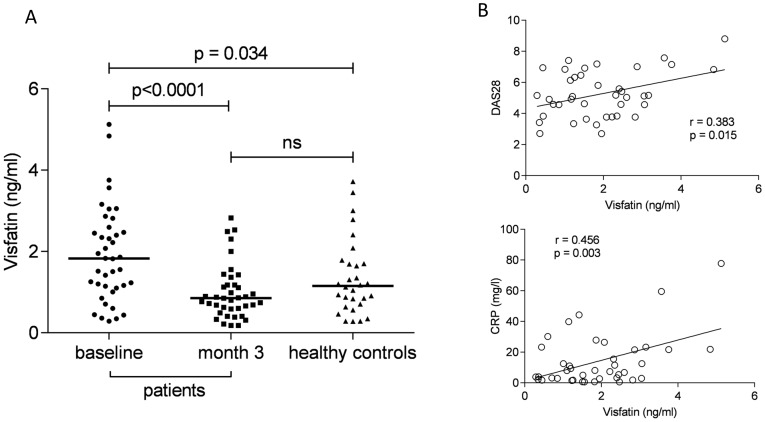
Comparison of serum visfatin levels between patients with early, treatment-naïve rheumatoid arthritis and healthy controls and change in serum visfatin after three months of treatment with conventional synthetic disease modifying antirheumatic drugs. (A). Association between baseline serum visfatin level and disease activity (B). CRP, C-reactive protein; DAS, disease activity score.

The baseline visfatin level in early RA patients was significantly higher compared to that in healthy controls (1.92±1.17 vs. 1.36±0.93 ng/ml; p = 0.034) (Fig. 1A). Using bivariate analysis, the visfatin level correlated positively with DAS28 (r = 0.383, p = 0.015) and CRP level (r = 0.456, p = 0.003) at baseline (Fig. 1B). The serum visfatin level was not related to age, gender or body mass index (BMI), but there was a negative association between visfatin and anti-CCP level (r = −0.400, p = 0.011) albeit not with IgM-RF (r = −0.045, p = 0.787).

### A decreased visfatin level is associated with disease activity improvement

The serum visfatin level significantly decreased after three months compared to baseline (1.92±1.17 vs. 0.99±0.67 ng/ml; p<0.0001) (Fig. 1A). Similar to baseline, after three months of treatment, the visfatin level correlated with DAS28 (r = 0.338, p = 0.035) and CRP (r = 0.588, p = 0.001).

**Figure 2:**
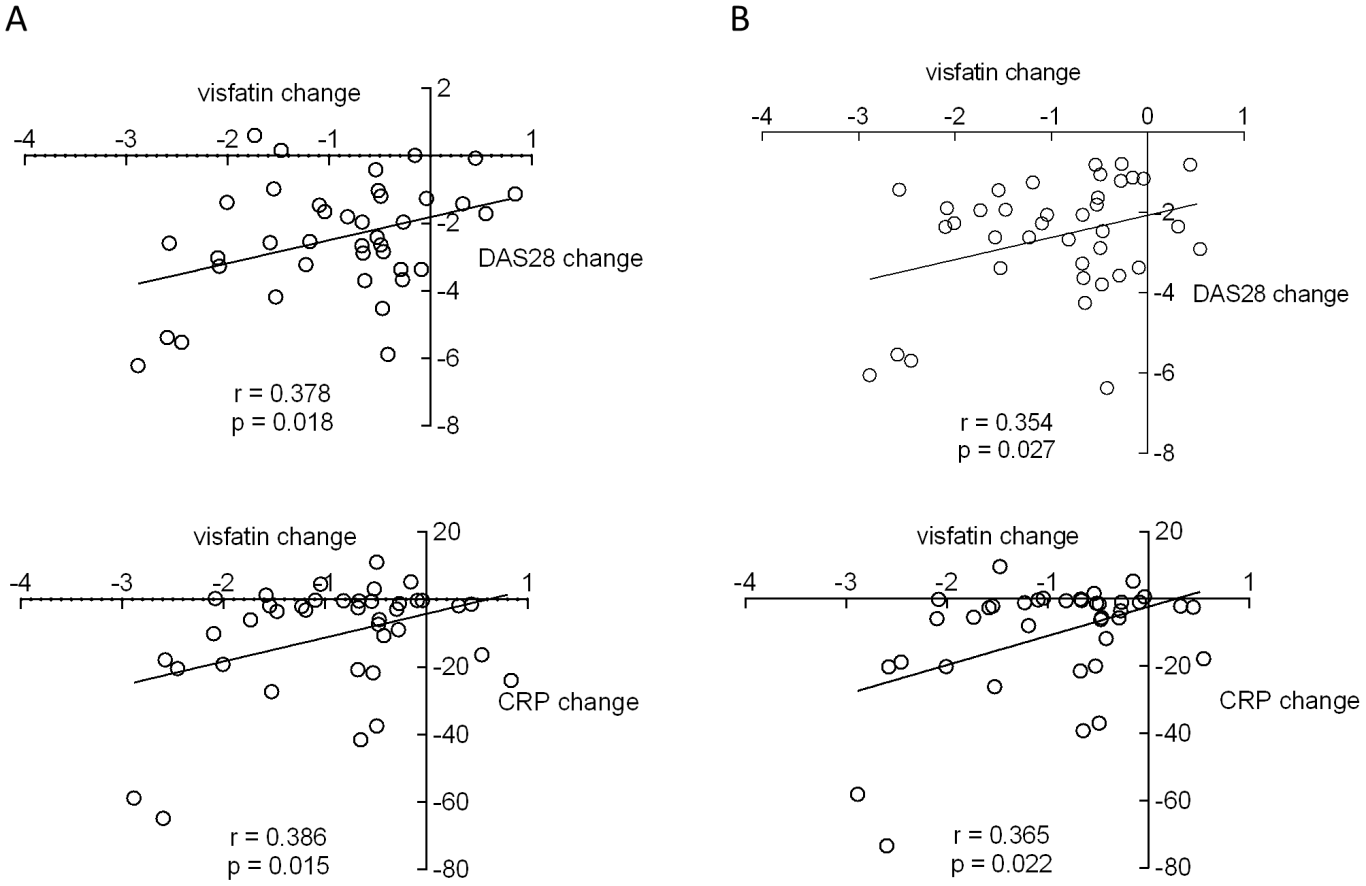
Correlations between visfatin reduction and disease activity improvement after three months (A) and between visfatin reduction after three months and disease activity improvement after 12 months (B). CRP, C-reactive protein; DAS, disease activity score.

A decrease in visfatin level correlated with a decrease in DAS28 (r = 0.378, p = 0.018) and CRP (r = 0.386, p = 0.015) after three months (Fig. 2A). Furthermore, a decrease in visfatin level after three months correlated with a decrease in DAS28 (r = 0.354, p = 0.027) and CRP (r = 0.365, p = 0.022) after 12 months (Fig. 2B). Using multiple linear regression analysis, a visfatin decrease after three months predicted DAS28 improvement between baseline and month three (p = 0.021, adjusted R^2^ = 11.9%) and between baseline and month 12 (p = 0.031, adjusted R^2^ = 10.1%).

### Visfatin level and lipid profile

The visfatin level did not correlate with serum lipids or atherogenic index at baseline in early RA patients (data not shown). While the mean total cholesterol (5.17±1.20 to 5.73±1.40 mmol/l; p<0.001) and HDL-cholesterol (1.07±0.25 to 1.32±0.29 mmol/l; p<0.001) significantly increased from baseline to month three, there was an improvement in the atherogenic index (4.02±1.35 to 3.50±1.29; p<0.01). Although a decrease in serum visfatin was not associated with an improved atherogenic index (r = 0.095, p = 0.567), the decrease was associated with an increase in total cholesterol (r = −0.328, p = 0.041). A decrease in visfatin significantly and independently predicted an increase in total cholesterol in all RA patients (p = 0.015). Another independent significant predictor in this model (adjusted R^2^ = 34.1%) was a decrease in CRP between baseline and month three (p = 0.007).

## Discussion

In this study, we have shown that an elevated visfatin level correlates with disease activity and that a decrease in visfatin level during the first three months of treatment independently predicts further disease activity improvement after 12 months in patients with treatment naïve early RA. The atherogenic index improved but was not related to visfatin reduction.

Consistent with previous studies demonstrating an increased visfatin level in patients with RA [3], [10], [14], we have shown that an elevated circulating visfatin in the early phase of the disease significantly decreased after three months of treatment. Because visfatin is up-regulated in several immune cells [3] and improvement in disease activity is associated with a decrease in macrophages in RA synovial tissue [16], a decrease in circulating visfatin may reflect a decrease in synovial immune cells following effective treatment. In addition, the visfatin level correlated with clinical and laboratory measures of disease activity at baseline and during treatment, which has been reported in some [3], [17], but not all, studies [12], [14]. This inconsistency may be explained by differences in populations and treatments because in patients with long-term and active RA on biological therapy, an association between visfatin and disease activity was not observed [12], [14].

Adipokines represent large group of highly bioactive substances secreted by adipocytes and immune cells that are involved in both metabolic and immunomodulatory functions. In addition to visfatin, Gonzalez-Gay et al demonstrated rapid reduction of serum resistin [18], but not adiponectin [19] levels in patients with long-term and active RA during anti-TNF-alpha therapy with infliximab. Resistin levels were positively associated with inflammatory markers of RA [18], whereas adiponectin levels were inversely associated with disease activity and low adiponectin levels clustered with metabolic syndrome features that reportedly contribute to atherogenesis in RA patients with severe disease [19].

We have found that a decrease in circulating visfatin correlates with disease activity improvement over the first three months of treatment. Furthermore, although baseline visfatin was not predictive, the change of visfatin level over the first three months predicted disease activity improvement after 12 months of treatment. Thus, a higher visfatin level in treated RA patients may be associated with more severe disease and thus higher risk of structural progression as demonstrated previously [11], [13]. These data further support a significant role for visfatin in the pathogenesis of RA.

There is evidence that a high visfatin level is associated with an increased risk of cardiovascular disease [20]. Klaasen et al. reported a relationship between a high visfatin level and an increased atherogenic index in adalimumab-treated RA patients and suggested that visfatin reduction can lead to decreased cardiovascular risk independent of disease activity [6]. However, in our study, the visfatin level correlated inversely with baseline total-cholesterol and its increase but not with atherogenic index. This finding may be explained by a different patient population and is consistent with a recent study by El-Hini et al. [20]. Because our patients had an improvement in atherogenic index during treatment [21], we suggest that the association between visfatin and total cholesterol level may be caused by dyslipidaemia, which occurs in early RA [21]. However, this hypothesis requires further investigation.

In conclusion, our study demonstrates an association between an elevated visfatin level and disease activity, and short-term reduction of visfatin is an independent predictor of long-term disease activity improvement in early, treatment-naïve RA patients. These results however need to be validated in larger and independent cohorts of patients with RA.
